# Functional disability and its associated factors among community- dweller older adults living in Gondar Town, Ethiopia: a community-based cross-sectional study

**DOI:** 10.1186/s12889-024-18110-y

**Published:** 2024-02-29

**Authors:** Mihret Dejen Takele, Getachew Azeze Eriku, Destaw Marie Merawie, Fiseha Sefiwu Zinabu, Molla Fentanew, Gashaw Jember Belay, Alemu Kassaw Kibret

**Affiliations:** Department of Physiotherapy, School of Medicine, College of Medicine and Health Sciences, University of Gondar, Gondar, Ethiopia

**Keywords:** Activities of daily living, Functional disability, Older adults, Ethiopia

## Abstract

**Background:**

Functional disability is an emerging public health concern that has an impact on the health and quality of life of older adults. If functional disability recognized early, it will be possible to support them to live independently. Although functional disability is extensively researched in developed countries; studies are scarce in Sub-Saharan Africa, particularly Ethiopia. Therefore, this study aims to assess the prevalence and associated factors of functional disability in activities of daily living among older adults in Gondar town, Ethiopia.

**Methods:**

A community-based cross-sectional study was conducted from April to June 2022. Multistage sampling techniques were used to recruit 607 older adults aged 60 years and older. A pre-tested interview-administered questionnaire was used to assess functional disability in basic activities of daily living and instrumental activities of daily living using the Katz Index and Lawton scale, respectively. Bivariate and multivariable logistic regression models were employed. The findings of the study were presented by descriptive statistics and an adjusted odds ratio with 95% CI was used to determine statistical significance.

**Results:**

The prevalence of functional disability in basic activities of daily living and instrumental activities of daily living among older adults was 34.5% and 54.4, respectively. Age 80 and older [AOR = 2.41, CI (1.41–4.10)], low-income status [AOR = 2.58, CI (1.50–4.46)], multimorbidity [AOR = 2.97, CI (1.92–4.60)], depression [AOR = 2.97, CI (1.63–5.40)], and low level of physical activity [AOR = 3.31, CI (2.11–5.17)] were associated with basic activities of daily living. Age 80 and older (AOR = 3.11, CI = 1.94-5.00), multimorbidity [AOR = 3.06, CI (2.10–4.46)], and depression [AOR = 3.52, CI (2.10–4.46)] were associated with instrumental activities of daily living.

**Conclusion and recommendations:**

Our study finding revealed that functional disability affects a large number of older adult residents. The age group of 80 years and older, low-income status, a low level of physical activity, multimorbidity, and depression were associated with basic and instrumental activities of daily living. Therefore, health interventions designed to increase older adults’ level of physical activity, management of multimorbidity, and depression, more care for elders 80 years and older, and supporting older adults financially for health insurance coverage could be an important strategy to reduce functional disability among older adults.

**Supplementary Information:**

The online version contains supplementary material available at 10.1186/s12889-024-18110-y.

## Introduction

Older individuals are wise and seasoned members of any society. Their knowledge, insight, and experience can help in the advancement of development, making them an invaluable resource for any country [1]. Despite their invaluable wisdom and insight, the aging of the world’s population is causing extensive economic and social consequences [2]. Globally the aging population has increased rapidly over the past few decades owing to two significant factors, namely, the reduction in mortality and fertility rates and improved quality of life, leading to an increase in life expectancy worldwide [3–5].

Global predictions indicate that the percentage of individuals over 60 will double, from 11 to 22%, between 2000 and 2050 [6]. Ethiopia had 5.2 million individuals over 60 in 2015, which is just more than 5% of the total population. By 2030 and 2050, the proportion of older Ethiopians will increase to 6.1% and 10.4%, respectively [7].

Functional disability is one of the major concerns among older people in the health system, leading to mortality and morbidity [8]. It is described as an acquired difficulty to carry out simple daily activities or more complicated tasks required for independent living [9]. It also frequently impairs functionality, which compromises the ability of an elderly person to perform activities of daily living (ADL) [10].

The most commonly used tools to measure functional disability are the Katz index of independence in activities of daily living and the Lawton instrumental activities of daily living scale. Katz index of independence in activities of daily living (ADL) uses the following six domains: bathing, dressing, toileting, transferring, continence, and feeding [11]. Instrumental activities of daily living scale (IADL) uses the following eight domains, such as using the telephone, shopping, preparing food, housekeeping, doing laundry, using transportation, handling medications, and handling finance [12].

According to estimates from the World Health Organization, the percentage of people with functional disabilities in developed countries is approximately 10.0%, whereas in developing countries it is approximately 15.0% [13]. According to a national survey, the prevalence of at least one ADL or IADL functional disability among older individuals 60 years of age and older was around 36.2% and 37.1% in the United States [14].

In Europe, the prevalence of at least one ADL disability ranged from 11 to 44%, while the prevalence of at least one IADL disability ranged from 8 to 40% [15]. Two rapidly aging countries, China and Japan, have stated that 20% of persons 65 years of age and beyond require long-term care due to a handicap; in 2015, the percentage of older adults in China with an ADL disability was 18.3% [16, 17].

Current evidence on prevalence of functional disability is unclear whether disability rates are rising or declining [18]. Although a decline in prevalence has been reported in some countries, an increase in aging population leads to an increasing of older adults with functional disabilities [19, 20]. Despite the fact that functional disability is an important public health issue, there is still insufficient data among older adults, especially in low- and middle-income countries.

According to the literature, functional disability in older adults may be associated with several individual factors [21], such as socioeconomic variables [22–25], the presence of diseases [22, 24, 25], and psychological factors [24, 26]. It is noteworthy that detecting these factors that have an influence on the functional ability of older people is essential to understand and implement prevention and intervention measures [21, 27, 28].

Impaired functional ability not only jeopardizes older people’s autonomy but also has an impact on the family, community, health system, and older adults themselves, increasing their vulnerability and dependence as they age, lowering their quality of life, and raising their risk of developing geriatric conditions like dementia, depression, incontinence, vertigo, falls, spontaneous bone fractures, and failure to thrive [29, 30].

However, the support for older people with functional disability needs to be comprehensive and involved with the community [31]. To the best of the author’s knowledge, there is no documented information about functional disability among the older adults in Ethiopia, despite the demographic transition of Ethiopia towards becoming an older society.

Thus, a comprehensive study of the prevalence of functional disability and its associated factors among the older adult population is needed, as people with this condition have greater needs for long-term care and healthcare. Therefore, the main aim of this study was to investigate the prevalence of functional disability and its associated factors among older adults living in Gondar town, Ethiopia.

## Methods and materials

### Study design and setting

From April to June 2023, a cross-sectional study based in the community was conducted. The study was conducted in the northwest Ethiopian town of Gondar, in the Amhara regional state. The city is found in the central Gondar zone of the Amhara regional state, about 180 km from Bahir Dar, the capital city of the Amhara regional state, and 748 km northwest of Addis Ababa, the capital city of Ethiopia. Among the nation’s most historical and highly populated cities is Gondar, which has a total of 25 kebeles (Ethiopia’s smallest administrative units) in the town of Gondar. Gondar town is a varied landscape, predominantly covered with a ragged hill and plateau formation, and with a variable temperature, it challenges especially older people with functional disabilities. The Gondar statistics agency projected that the total population of Gondar town would be 390,000 in 2021/22, based on data from the 2007 population census. Of this total, women represented over half of the population, and 6879 of these were older adults [32]. Older adults in Ethiopia play a key role in contributing to the social and economic fabric of the family. They are wise and seasoned members of any society; their knowledge, insight, and experience can help in the advancement of development, making them an invaluable resource for the country. However, older people in Gondar town are facing many challenges, including health problems, lack of shelter, unsuitable residential areas, the absence of family and community support, the absence of education and training opportunities, and limited employment and income-generating opportunities [33]. The town has one comprehensive specialized hospital and eight health centers; they are providing health services to the population.

### Source population

The source population included all Gondar town permanent residents who were 60 years of age or older and lived in communities. The study population consisted of older adults who were 60 years of age or older and living in particular kebeles, which are Ethiopia’s smallest administrative units, during the study period. Older adults—those who were 60 years old or older and had lived in the chosen kebeles for at least six months on a permanent basis—were included.

### Sample size and sampling procedure

The sample size was determined using a single population proportions formula, assuming a 50% anticipated prevalence of functional disability since there was no study conducted before in Ethiopia, a 95% confidence interval, and a 5% marginal error. The minimum sample size (n) required for the study was calculated by using a design effect of 1.5 and a 10% non-response rate. With this, the total sample size was found to be 636. The multi-stage sampling technique was used to select participants with the assumption of a homogenous population. The town had six sub-cities, each composed of different kebeles (the smallest administrative units in Ethiopia). Three sub-cities were selected by the lottery method and clustered into 25 kebeles. 30% percent of the kebeles and their households were also selected by the lottery method based on administrative record numbers, and every household in the selected kebeles was visited to interview participants.

### Variables

#### Dependent variables

Functional disability (yes / no).

#### Independent variables

Sex, age, religious, marital status, educational level, living arrangement, employment status, income status, hospitalization, multimorbidity, depression, **s**moking, alcohol users, and physical exercise.

### Operational definition

#### Older age

Those participants who are older than or equal to 60 years old were considered as older age people [34].

#### Functional disability

Defined as acquired difficulty in performing basic everyday tasks or more complex tasks needed for independent living [9]. It was measured by the **Katz Index of Independence** the index ranks the adequacy of performance in the six functions of bathing, dressing, toileting, transferring, continence, and feeding. Older adults are scored yes or no for independence in each of the six functions. A score of 6 indicates full function, and a score less than 6 indicate having functional disabilities [11]. The other types of functional disabilities were measured by t**he Lawton IADL scale.** There are eight domains of function measured, such as telephone, shopping, preparing food, housekeeping, doing laundry, using transportation, handling medications, and handling finances. Due to the culture of work, men have not been rated in the areas of food preparation, housekeeping, and laundry. Women are assessed in all eight areas of function. Scores for older persons are based on their maximum functioning level within that category. A summary score ranges from 0 (low function, dependent) to 8 (high function, independent) for women and 0 through 5 for men [12].

#### Depression

The older persons were assessed using 15 items of the Geriatric Depression Scale Short Form (GDS-SF), with a cut point of greater than or equal to five. If they scored five or above, they were considered depressed; if they scored below five, they were not [35, 36].

#### Physical exercise

Is any kind of moderate– intensity exercise (such as walking, cycling, sports or planned exercise, and strength exercise) done for at least 150 min per week [37].

#### Smoker

Those who smoke any tobacco products daily were considered tobacco users [38].

#### Multimorbidity

Coexistence of two or more chronic conditions in older adults [39].

#### Hospitalizations

Any history of visiting hospitalization in past one year among older adults [40].

#### Alcohol user

Those who consumed any type of alcohol 2 or more bottles daily was considered as alcohol users [41].

### Data collection methods and tools

After permission obtained from the Ethical review committee of the University of Gondar collage of medicine and health sciences, house to house visit was done. Participants in the study were interviewed face-to-face using a pre-tested, predesigned structured schedule encompassing sociodemographic and economic variables such as sex, age, religion, marital status, educational attainment, type of housing, employment status, and income status. Health related variables like hospitalization, multimorbidity, depression, functional disability and life style and behavioral variable like physical exercise, alcohol user and smoker were also assessed. The questionnaire was derived from previous research, and it was modified to meet the objective of the study. To ensure consistency, it was first prepared in an English language and then translated by language experts into Amharic (local language), then back into English. Data collection was done by four trained health extension workers and two physiotherapist supervisors. To ensure the quality of the data, all data collectors and supervisors were given one-day training prior to the actual data collection by the principal investigator on the purpose of the study, details of the data collection instrument (questionnaire), interviewing techniques, importance of privacy and insuring confidentiality of the respondents. The supervisor checked for the completeness and consistency of the data. The data collection tools were also pre-tested on 5% of the total sample size before the actual data collection period to check for the accuracy of responses, language clarity, and appropriateness of the tools. For the actual study, the necessary change was made after pretest.

### Statistical analysis

The collected data was first coded and entered into Epidata and then exported into Statistical Package for the Social Sciences (SPSS) Version 26 for analysis [42]. Binary and multivariable logistic regression analysis was done to identify the factors associated with the outcome variable. Prior to determining the final independent predictor variables for ADLs and IDL, the bivariate logistic regression analysis was done, and all variables that were determined to be statistically significant at *P*-value < 0.25 were included together in the multiple logistic regression analysis. Variables with a *P* value of < 0.05 at 95% confidence interval (CI) and adjusted odds ratio (AOR) were used to interpret the findings of the final model.

## Results

A total of 607 older adults were included in this study, giving a response rate of 95%. Among the total respondent population, (51.4%) were male, (37.9%) were aged between 60 and 69 years, (75.9%) were of the Orthodox Christian religion, (50.6%) had secondary educational status, (67.2%) were married, (38.1%) were retired, (40.7%) lived with their spouse only, and (40.9%) had a monthly income of ≤ 1500 ETB. Table 1 shows the frequency distribution of sociodemographic and economic variables.

**Table 1:** Frequency distribution of sociodemographic and economic variables among community dweller older adults living in Gondar town, Northwest, Ethiopia, 2023 (*n* = 607)

Variables	Overall		Male		Female	
	N	%	n	%	N	%
**Age in years**						
60–69	230	37.9	135	22.2	95	15.7
70–79	151	24.9	76	12.5	75	12.4
80 and above	226	37.2	101	16.6	125	20.6
**Religions**						
Orthodox Christian	459	75.6	235	38.7	224	36.9
Muslim	97	16	51	8.4	46	7.6
Protestant	37	6.1	17	2.8	20	3.3
Jewish	14	2.3	9	1.5	5	0.8
**Educational status**						
Illiterate or Primary	91	15.0	28	4.6	63	10.4
Secondary	307	50.6	165	27.2	142	23.4
Tertiary or higher	209	34.4	119	19.6	90	14.8
**Marital status**						
Unmarried/divorce/widow	199	32.8	78	12.9	121	19.9
Married	408	67.2	234	38.6	174	28.7
**Employment status**						
Salaried	141	23.2	85	14	56	9.2
Self-employed	200	32.9	105	17.3	95	15.7
Retired with pension	231	38.1	111	18.3	120	19.8
Currently not working	27	4.4	9	1.5	18	3.00
Never work for money	8	1.3	2	0.3	6	1
**Living arrangement**						
Living with spouse only	247	40.7	146	24.1	101	16.6
Living with children/other family	215	35.4	97	16	118	19.4
Living alone	145	23.9	69	11.4	76	12.5
**Monthly individual income in ETB**						
≤ 1500	291	47.9	130	21.4	161	26.5
1501–3500	112	18.5	66	10.9	46	7.6
≥ 3501	204	33.6	116	19.1	88	14.5

### Prevalence of functional disability

The prevalence of functional disability in **ADL** was 34.5% (CI 95%, 30.5–38.2), whereas the prevalence of functional disability in IADL was 54.4% (CI 95%, 50.4–58.5). In this study, the prevalence of functional disability in each basic functional activity was as follows: the majority of the study participants had 62 (10.9%) bathing difficulties, 39 (6.4%) toileting difficulties, and in instrumental activities of daily living, 107 (62.6%) transportation difficulties, and 62 (10.2%) shopping difficulties. See Table 2 for more details.

**Table 2:** Frequency distribution of specific domain functional disabilities in both ADLs and IADL

Variables	ADLs disabilities		IADL disabilities		
	N	%	Variables	n	%
Independent	400	65.9	Independent	272	44.8
Bating	62	10.2	Using the telephone	19	3.1
Dressing	36	5.9	Shopping	62	10.2
Toileting	39	6.4	Preparing food	28	4.6
Transfer	30	4.9	House keeping	31	5.1
Continence	20	3.3	Doing laundry	53	8.7
Feeding	20	3.3	Handling medication	21	3.5
			Handling finance	14	2.3
			Using transportation	107	17.6

### Factors associated with functional disability in ADL and IADL

Regarding the associated factors, the variables of the preliminary bivariable analysis submitted to multivariable analysis, according to the inclusion criteria (*P* < 0.2) include: age 80 years and above, lower income status, having multimorbidity, depression, and a lower physical activity level, were associated with ADLs. In terms of functional disability in IADL, the associated factors were age 80 and above, multimorbidity, and symptoms of depression. The variables included in the multivariate logistic regression model are shown in Tables 3 and 4.

**Table 3:** Final logistic regression model for the variables associated with functional disability in ADLs among community dweller older adults living in Gondar town, Northwest, Ethiopia, 2023 (*n* = 607)

Functional disability in ADLs			OR (95%CI)		
Variables	Yes	No	COR (95%CI)	AOR (95%CI)	*P*-Value
**Gender**					
Male	85	227	1	1	
Female	123	172	1.91(1.35–2.68	1.28(0.82–1.99)	0.27
**Age in years**					
60–69	43	187	1	1	
70–79	38	113	1.46(0.92–2.33)	0.71(0.38–1.32)	0.28
80 and older	127	99	5.57(3.65–8.51)	2.41(1.41–4.10)	0.01^*^
**Educational status**					
Primary or less	46	45	3.00(1.79–5.03)	1.44(0.72–2.8)	0.29
Secondary	109	198	1.62(1.09–2.39)	0.98(0.59–1.63)	0.96
Tertiary or higher	53	156	1	1	
**Marital status**					
Married	94	314	1	1	
Unmarried/divorce/widowed	114	85	4.44(3.11–6.44)	1.25(0.73–2.12)	0.40
**Living arrangement**					
Living with spouse	38	209	1	1	
Living with children /other family	96	119	4.43(2.86–6.87)	2.10(1.28-4.0)	0.05
Living alone	74	71	5.73(3.56–9.21)	2.53(1.31–4.88)	0.06
**Monthly Income status in ETB**					
≥ 3501	32	172	1	1	
1500–3500	25	87	1.54(0.86–2.76)	1.41(0.70–2.81)	0.32
< 1500	151	140	5.76(3.72–9.01)	2.58(1.50–4.46)	0.01^*^
**Hospitalizations**					
No	136	310	1	1	
Yes	72	89	1.84(1.27–2.67)	1.60(0.98–2.62)	0.05
**Multimorbidity**					
No	77	262	1	1	
Yes	131	132	3.25(2.29–4.61)	2.97(1.92–4.60)	0.001^**^
**Depression**					
No	19	146	1	1	
Yes	189	253	5.74(3.43–9.59)	2.97(1.63–5.40)	0.001^**^
**Physical activity level (in minutes per week)**					
≥ 150	82	302	1	1	
< 150	126	97	4.78(3.33–6.85)	3.31(2.11–5.17)	0.001^**^
**Smoking status**					
No	187	369	1	1	
Yes	21	30	1.38(0.77–2.47)	1.11(0.49–2.53	0.79
**Alcohol consumption**					
No	166	299		1	
Yes	42	100	0.75(0.50–1.13)	0.99(0.57–1.73)	0.99

COR: Crude Odds ratio; AOR: Adjusted Odd ratio; CI: Confidence interval; ETB: Ethiopian birr; ADLs: Basic activity of daily living * *P* < 0.05, ** *P <* 0.001, 1: reference category

**Table 4:** Final logistic regression model for the variables associated with functional disability in IADL among community dweller older adults living in Gondar town, Northwest, Ethiopia, 2023 (*n* = 607)

Functional disability in IADL			OR (95%CI)		
Variables	Yes	No	COR (95%CI)	AOR (95%CI)	*P*-Value
**Gender**					
Male	155	157	1	1	
Female	176	119	1.49(1.08–2.06)	1.26(0.86–183)	0.22
**Age in years**					
60–69	96	134	1	1	
70–79	70	81	1.20(0.79–1.82)	1.00(0.61–1.62)	0.99
80 and older	165	61	3.77(2.54–5.59)	3.11(1.94-5.00)	0.001^**^
**Educational status**					
Primary	56	35	1.38(0.83–2.29)	0.83(0.45–1.53)	0.55
Secondary	163	144	0.98(0.68–1.39)	0.65(0.42–0.99)	0.48
Tertiary or higher	112	97	1	1	
**Marital status**					
Married	201	207	1		
Unmarried/divorce/widowed	130	69	1.94(1.36–2.75)	0.92(0.56–1.51)	0.75
**Living arrangement**					
With spouse	111	136	1	1	
Living with children/family	132	83	1.94(1.34–2.82)	1.52(0.95–2.45)	0.8
Living alone	88	57	1.89(1.24–2.87)	1.36(0.78–2.37)	0.27
**Monthly income in ETB**					
≥ 3501	99	105	1	1	
1500–3500	58	54	1.13(0.71–1.80)	1.20(0.69–2.07)	0.51
< 1500	174	117	1.57(1.09–2.26)	0.72(0.45–1.15)	0.17
**Hospitalization**					
No	241	205	1	1	
Yes	90	71	1.07(0.75–1.54)	0.88(0.57–1.35)	0.56
**Multimorbidity**					
No	144	195			
Yes	187	81	3.12(2.22–4.38)	3.06(2.10–4.46)	0.001^**^
**Depression**					
No	49	116	1	1	
Yes	282	160	4.17(2.83–6.13)	3.52(2.27–5.45)	0.001^**^
**Physical activity level (in minute per week)**					
≥ 150	183	201	1	1	
< 150	148	75	2.16(1.53–3.05)	1.45(0.97–2.17)	0.70
**Smoking status**					
No	302	254			
Yes	29	22	1.10(0.62–1.97)	0.48(0.48–198)	0.98
**Alcohol consumption**					
No	261	204	1	1	
Yes	70	72	0.76(0.52–1.10)	0.92(0.58–1.45)	0.72

COR: Crude odds ratio; AOR: Adjusted odds ratio; CI: Confidence interval; ETB: Ethiopian Birr; IADL: Instrumental activity of daily living; * *P* < 0.05, ** *P <* 0.001, 1: reference category

## Discussion

The aim of this study was to assess the prevalence and associated factors of functional disability among community dwellers older adults living in Gondar town. In this study we found that functional disability affects a large number of older adult residents in Gondar town. The prevalence of functional disability in ADL among older adults was found to be 34.5% (CI 95%, 30.5–38.2) and 54.4% (CI 95%, 50.4–58.5) older adults lived with IADL functional disability. The findings of the present study revealed that functional disability in ADL among older adults was found to be associated with age (80 years and older), low-income status, multimorbidity, symptoms of depression, and low level of physical activity, whereas age 80 and older, having multimorbidity and symptoms of depression were associated with IADL functional disability.

The prevalence of functional disability in our study was in concordance with the results obtained in other studies done in Spain (34.6%) on ADL and (53.5%) on IADL [43], Sweden (32%) [44] on ADL, India (37.5% ) [45], and Nigeria (30%) [46]; this might be due to a similar study measurement tool and the mean age of the study participants. For instance, similar to our studies, researches conducted in Spain and Sweden used similar functional disability assessment tool for ADL and IADL, and the mean age of the study participants in studies done in Nigeria (with a mean age of participants 69.78 ± 6.69 years (age range = 60–98 years)) and India (participants age above 60 years with mean age 67.8 ± 7.41 years), was closely comparable to that of our study. On the other hand, two studies conducted in Northern India (43%, 44%) [47, 48], Ghana (55%) [49], South Korea (63%) [50] showed higher percentages of functional disability when compared to our study.

The higher prevalence of functional disability in ADL compared to IADL is in agreement with the studies conducted in the Brazilian state of Minas (78.3%, 21.4%) [51], Saudi Arabia, Riyadh (58.8%, 24%) [52], Brazil, Bagé (30%, 10%) [53], and Uberaba, Brazil (65.9%, 21.2%) [6]. However, studies conducted in Pelotas (28.8%), Porto Alegre (26.1%), and Montes Claros (25.9%) showed prevalence rates of disability in IADL lower than our study [6, 25, 54]. Differences in the prevalence rates in ADL and IADL occur because of the different domain used and the socioeconomic differences between the regions. In addition, IADL has a wider variety of activities and the impact that would have on disability estimates.

The finding of the present study found that functional disability increases with rising age, and older adults had higher odds of having functional disability than younger adults. According to our study, those who were ≥ 80 years of age were 2.4 times more likely have ADL functional disability and 3.1 times more likely to have IADL functional disability as compared to younger age groups (60–69 years) which is in agreement with previous studies conducted [6, 43, 47, 53]. This might be due to being older age is a major risk factor for the deterioration of functional ability in older individuals [54]. This is because the ability to accomplish a task requires the integration of numerous physiological systems, and these systems often deteriorate with age [55].

The results of this study also showed that older adults with a low income status were 2.5 times more likely to have functionally disability in ADL compared with their counterparts, which is in agreement with other studies [46, 56–58]. The possible reason might be older people with low income status have deteriorated health conditions in early life stages, resulting in persistent health inequality in old age [59]. Furthermore, life time disadvantages could make poor older adults more vulnerable to health problems and require more money for medical treatment [60].

This study also showed that older people with multimorbidity are 2.9 times more likely to have ADL disability and 3.0 times more likely to have IADL disability compared with their counterparts, which is supported by previous studies [6, 43, 53, 61]. This happens as a consequence of the fact that chronic illnesses might be connected to a decline in functional abilities [25]. In addition, the higher number of diseases often leads to a decrease in quality of life and social isolation, thus resulting in functional disability. However, having a disease doesn’t necessarily mean showing impaired performance of daily tasks [62]. It should be noted that the presence of various diseases is a challenge of the aging process, a situation that may contribute to difficulties in activities of daily living because of the impact on independence and autonomy [6].

Furthermore, this study found that older people with low levels of physical activity were 3.3 times more likely to have ADL disability compared with their counterparts in line with other studies [63–65]. This is might be that older adults with a varying level of disabilities who had previously reported engaging in at least some sort of physical activity experienced a slower rise in their functional limitations [64]. By influencing changes in functional limitations, this low level of physical exercise may decrease the progression of ADL impairments [66]. In addition, a systematic review suggested that older people who were more physically active or who did regular exercise had a lower risk of having ADL functional disability [67].

Regarding to the symptom of depression our result found that older adults with depression were 2.9 times more likely to have ADL functional disability and 3.5 times more likely to have IADL disability compared with older adults not depressed similar to studies conducted in Brazil, India, and China [6, 48, 51, 68]. The correlation seen between declining physical function and cognitive impairment could perhaps be attributed to differences in the onset of depression [69]. The mechanism for this cycle remains unknown; however, some behavioral and biological conditions are able to negatively influence functional ability [26]. In addition, depressive symptoms may have a negative impact on the quality of life and social relations of the elderly [24].

## Conclusion

This study showed that functional disability among older adults in Gondar town, Ethiopia, was 34.5% in ADL and 54.4% in IADL. Finding of this study have shown that age group of 80 years and older, low-income status, low level of physical activity, multimorbidity, and depression were significantly associated with ADL functional disability. In addition, age group of 80 years and older, multimorbidity, and depression were significantly associated with IADL functional disability. Therefore, health interventions designed to increase older adults’ level of physical activities, management of multimorbidity, depression, more care for elders 80 years and older and supporting older adults financially for health insurance coverage could be an important strategy to reduce functional disability among older adults. In addition, this study can be considered as an early warning and advice to health professionals like physiotherapy, occupational therapy, health policymakers, and other pertinent stakeholders to take effective control measures, conduct periodic assessments, and identify contributing factors to functional disability among older adults in the study area.

### Strength and limitations of the study

This study has some limitations. First, due to the nature of a cross-sectional study, it is difficult to make a causal inference. Second, because we only included older adults who lived in metropolitan areas—whose lifestyles differ from those of rural communities—our study’s conclusions are unlikely to apply in other circumstances. Thirdly, the measures of functional limits (ADLs and IADL) are self-reported, which may further pose issues with statistical endogeneity because of the extensive study of the aging population in our study area. Despite the limitations, to the best of the authors’ knowledge, this is the first study of its kind to look at functional disability in older individuals, conducted in Gondar, Ethiopia. Besides, because the study was conducted in a community to determine the prevalence of functional disability and associated factors among older adults, it may accurately reflect the actual nature of the issue.

### Electronic supplementary material

Below is the link to the electronic supplementary material.

Supplementary Material 1

## Data Availability

Since this is a funded work, the raw data is the property of the University of Gondar. The datasets used and/or analyzed during the current study are available from the corresponding author on reasonable request at **mihretdejen2017@gmail.com**.

## Abbreviations

AOD: Adjusted Odd Ratio
ADL: Activity of Daily Living
CI: Confidence Interval
COR: Crude Odd Ratio
GDS: SF-Geriatric depression scale short form
IADL: Instrumental activity of daily life
SPSS: Statistical Package for Social Software
WHO: World Health Organization
