# Supplementary material for: Functional disability and its associated factors among community- dweller older adults living in Gondar Town, Ethiopia: a community-based cross-sectional study
Source: BMC Public Health. 2024 Feb 29;24:647. doi: 10.1186/s12889-024-18110-y (PMC10905928; doi:10.1186/s12889-024-18110-y)
Supplement: Supplementary file 1 — Supplementary Material 1 [file 12889_2024_18110_MOESM1_ESM.docx]

Fill or circle the space that provide your answer

| **Part one: Scio demographics information** | | | |
| --- | --- | --- | --- |
| No | Question | Possible answer | skip |
| 101 | What is the sex of the respondent? | 1. Male 2. Female |  |
| 102 | Ho w old are you? | ________________ in year |  |
| 103 | What is your marital status? | 1. Unmarried 2. Marred 3. Divorce 4.Widow |  |
| 104 | What is your education level? | 1. None 2. primary education 3. Secondary education. 4. Higher professional or university education |  |
| 105 | How is your living arrangement | 1. Living alone 2.Living with children/ others family 3.With spouse only |  |
| 106 | What is your current monthly income in Ethiopian birr (ETB)? | ---------birr cent------ |  |

|  | **Part two: Clinical related factors** |  | |  |
| --- | --- | --- | --- | --- |
| 201 | Do you have chronic disease? | 1.Yes | 2.No | HF1 |
| 202 | If you say yes question HF1 how many chronic disease do you have? | ___________________________ | |  |
| 203 | Do you have a history hospitalization in past one year? | 1.Yes | 2.No |  |

|  | **Part three: Measuring activity of daily life by using Katz Index of Independence** | | |  |
| --- | --- | --- | --- | --- |
|  | Activities |  | |  |
| 301 | Do you ask supervision, direction or personal assistance when you bathing? | 1.Yes | 2.No |  |
| 302 | Do you ask supervision, direction or personal assistance when you dressing? | 1.Yes | 2.No |  |
| 303 | Do you ask supervision, direction or personal assistance when your toileting? | 1.Yes | 2.No |  |
| 304 | Do you asking supervision, direction or personal assistance when you move and out of bed or moving from bed to chair? | 1.Yes | 2.No |  |
| 305 | Can you control when your urine or bowels are coming out until you reach the toilet? | 1.Yes | 2.No |  |
| 306 | Do you move food from plate into mouth without help? | 1.Yes | 2.No |  |

| **Part four:** Lawton - Brody Instrumental Activities Of Daily Living Scale (I.A.D.L.) | | | |
| --- | --- | --- | --- |
| Scoring: For each category, circle the item description that most closely resembles the client’s highest functional level (either 0 or 1). | | | |
| A. Ability to Use Telephone |  | E. Laundry | |
| 1. Operates telephone on own initiative-looks up and dials numbers, etc.  2. Dials a few well-known numbers  3. Answers telephone but does not dial  4. Does not use telephone at al | 1  1  1  0 | 1.Does personal laundry completely  2. Launders small items-rinses stockings, etc.  3. All laundry must be done by others | 1  1  0 |
| B. Shopping |  | F. Mode of Transportation |  |
| 1. Takes care of all shopping needs independently  2. Shops independently for small purchases 3. Needs to be accompanied on any shopping trip  4. Completely unable to shop | 1  0  0  0 | 1. Travels independently on public transportation or drives own car  2. Arranges own travel via taxi, but does not otherwise use public transportation  3. Travels on public transportation when accompanied by another  4. Travel limited to taxi or automobile with assistance of another  5. Does not travel at all | 1  1  1  0  0 |
| C. Food Preparation |  | G.Responsibility for Own Medications |  |
| 1. Plans, prepares and serves adequate meals independently  2. Prepares adequate meals if supplied with ingredients  3. Heats, serves and prepares meals, or prepares meals, or prepares meals but does not maintain adequate diet  4. Needs to have meals prepared and served | 1  0  0  0 | 1. Is responsible for taking medication in correct dosages at correct time  2. Takes responsibility if medication is prepared in advance in separate dosage  3. Is not capable of dispensing own medication | 1  0  0 |
| D. Housekeeping |  | H. Ability to Handle Finances |  |
| 1. Maintains house alone or with occasional assistance (e.g. "heavy work domestic help") 2. Performs light daily tasks such as dish washing, bed making  3. Performs light daily tasks but cannot maintain acceptable level of cleanliness  4. Needs help with all home maintenance tasks  5. Does not participate in any housekeeping tasks | 1  1  1  1  0 | 1. Manages financial matters independently (budgets, writes checks, pays rent, bills, goes to bank), collects and keeps track of income  2. Manages day-to-day purchases, but needs help with banking, major purchases, etc.  3.Incapable of handling money | 1  1  0 |
| Score |  | score |  |
| Total score____________  A summary score ranges from 0 (low function, dependent) to 8 (high function, independent) for women and 0 through 5 for men to avoid potential gender bias. | | | |

|  | **Part four: Measurement of depression using GDS-SF** |  |  |  |
| --- | --- | --- | --- | --- |
| 401 | Are you basically satisfied with your life? | 1.Yes | 2.No |  |
| 402 | Have you dropped many of your activities and interests? | 1.Yes | 2.No |  |
| 403 | Do you feel that your life is empty? | 1.Yes | 2.No |  |
| 404 | Do you often get bored? | 1.Yes | 2.No |  |
| 405 | Are you in good spirits most of the time? | 1.Yes | 2.No |  |
| 406 | Are you afraid that something bad is going to happen to you? | 1.Yes | 2.No |  |
| 407 | Do you feel happy most of the time? | 1.Yes | 2.No |  |
| 408 | Do you often feel helpless? | 1.Yes | 2.No |  |
| 409 | Do you prefer to stay at home, rather than going out and doing new things? | 1.Yes | 2.No |  |
| 410 | Do you feel you have more problems with memory than most? | 1.Yes | 2.No |  |
| 411 | Do you think it is wonderful to be alive now? | 1.Yes | 2.No |  |
| 512 | Do you feel pretty worthless the way you are now? | 1.Yes | 2.No |  |
| 413 | Do you feel full of energy? | 1.Yes | 2.No |  |
| 414 | Do you feel that your situation is hopeless? | 1.Yes | 2.No |  |
| 415 | Do you think that most people are better off than you are? | 1.Yes | 2.No |  |

|  | **Part five : Information on lifestyle factors** | | | |
| --- | --- | --- | --- | --- |
| 501 | Have you engaged in any type of regular physical exercise (Aerobic exercise, muscle strengthening exercise and balance exercise). | 1.Yes | 2.No | BF01 |
| 502 | If you say yes question BF01 how often do you do exercise per week | ……………….. minute/day  ………………..day/week | | |
| 503 | Do you smoke cigarette | 1.Yes | 2.No | BF03 |
| 504 | If you answer yes for BF03, how many cigarettes do you smoke per day? | ________Cigarette per day |  |  |
| 505 | Do you drink alcohol? | 1.Yes | 2.No | BF05 |
| 506 | If you answer yes for BF05, How much alcohol drink per day | 1. ≥ 2 bottle beer/day 2. < 2 bottle beer/day | | |
